# NURSE PERCEPTIONS OF A SYSTEMS-BASED OLDER ADULT MOBILITY INTERVENTION IN ACUTE CARE UNITS

**DOI:** 10.1093/geroni/igae098.4032

**Published:** 2024-12-31

**Authors:** Olivia Erickson, David Tucker, Barbara King, Linsey Steege

**Affiliations:** University of Wisconsin-Madison, Madison, Wisconsin, United States; University of Wisconsin-Madison, Madison, Wisconsin, United States; UW-Madison, Madison, Wisconsin, United States; University of Wisconsin-Madison, Madison, Wisconsin, United States

## Abstract

Lack of ambulation during a hospital stay is the leading cause of hospital-acquired disability for older adult patients. Acute care nursing staff are identified as responsible for maintaining patient functional ability. However, nurses infrequently walk patients due to system barriers. Multiple interventions to improve patient ambulation have been tested with mixed results. Few researchers have designed interventions that target nurse barriers or addressed factors that may impact nurse acceptance of a mobility intervention or sustainability. This study aimed to understand how nurses experienced implementing a systems-based mobility intervention and identify which components of the intervention need further adaptation to create the right “fit” for inpatient units. A qualitative study using directed content analysis was conducted. Four adult medical inpatient units from 2 academic medical centers in the Midwest served as the study sites. 53 staff members participated in 8 focus groups that were audio recorded and transcribed. Four categories that describe the nursing staff experience were identified: Needing the Right Dose, Fostering Communication, Being Valued, and Making Ambulation Visible. Nurses perceived the intervention as adaptable to fit their unit needs, improved communication between staff members, patients, and healthcare providers, and aligned with their value of achieving good patient outcomes. It is critical to understand nurses’ experiences with implementing a systems-based intervention when promoting behavior change to improve older adult patient ambulation. Failed implementation efforts can have serious consequences on nurses’ perceptions of patient ambulation, the sustainability of programs, and the quality of care for older adults.

